# Development and Characterization of Cyclodextrin-Based Nanogels as a New Ibuprofen Cutaneous Delivery System

**DOI:** 10.3390/pharmaceutics14122567

**Published:** 2022-11-23

**Authors:** Marzia Cirri, Giulia Nerli, Natascia Mennini, Francesca Maestrelli, Paola Mura

**Affiliations:** Department of Chemistry, University of Florence, Via Schiff 6, Sesto Fiorentino, 50019 Florence, Italy

**Keywords:** nanogel, soluble β-cyclodextrin/epichlorohydrin polymer, Carbopol, hydroxypropyl-β-cyclodextrin, hydroxypropyl methylcellulose, ibuprofen, improved topical delivery

## Abstract

Nanogels combine the properties of hydrogels and nanocarrier systems, resulting in very effective drug delivery systems, including for cutaneous applications. Cyclodextrins (CDs) have been utilised to enhance the nanogels’ loading ability towards poorly soluble drugs and promote/sustain drug release. However, formation of CD-based nanogels requires the use of specially modified CDs, or of crosslinking agents. The aim of this work was to develop a CD-based nanogel to improve the cutaneous delivery of ibuprofen by using the soluble β-cyclodextrin/epichlorohydrin polymer (EPIβCD) without adding any potentially toxic crosslinker. The use of EPIβCD enabled increasing ibuprofen loading due to its complexing/solubilizing power towards the poorly soluble drug and prolonging drug release over time due to the nanogel formation. DLS analysis proved that EPIβCD allowed the formation of nanostructures ranging from 60 up to 400 nm, depending on the gelling agent type and the gel preparation method. EPIβCD replacement with monomeric HPβCD did not lead in any case to nanogel formation. Permeation experiments using skin-simulating artificial membranes proved that the EPIβCD-based nanogel enhanced ibuprofen solubility and release, increasing its permeation rate up to 3.5 times, compared to a reference formulation without CD and to some commercial gel formulations, and also assured a sustained release. Moreover, EPIβCD replacement with HPβCD led to a marked increase in drug solubility and initial release rate, but did not provide a prolonged release due to the lack of a nano-matrix structure controlling drug diffusion.

## 1. Introduction

Ibuprofen (2-4(4-isobutylphenyl) propionic acid) is one of the best known nonsteroidal anti-inflammatory drugs (NSAIDs). It is widely used as an analgesic, anti-inflammatory, and antipyretic agent, mainly in the treatment of mild to moderate pain related to dysmenorrhea, headache, postoperative dental pain, soft tissue injury, rheumatoid and osteoarthritis [1,2]. However, frequent oral use of this drug, as for other NSAIDs, can cause several adverse effects, especially against the upper gastrointestinal tract (gastric ulcers, nausea, vomiting) [3], but also at the cardiovascular and renal levels. It has been reported that 90% of the patients treated orally with NSAIDs were at risk of gastrointestinal adverse events and 44.3% were at risk of adverse cardiovascular events [4,5].

Therefore, the development of topical and/or transdermal dosage forms of ibuprofen allowing to both avoid gastrointestinal adverse effects and limit other side effects observed after oral administration as well as to provide consistent drug levels at the application site for prolonged time is considered very desirable, particularly in the treatment of local inflammatory pain, such as muscle aches and arthritis. A recent review proved the increasing interest in the topical use of NSAIDs to treat musculoskeletal conditions and obtain pain relief in both acute (such as sprains, strains, and overuse injuries) or chronic (osteoarthritis, neuropathic pain) conditions [6]. It has been shown that the formulation plays a critical role in the therapeutic efficacy of topical drug delivery [6]. This is particularly true in the case of ibuprofen due to its low water solubility and poor skin permeability, which can give rise to variable/unpredictable transdermal delivery, as proved by the very different skin permeation profiles obtained when comparing different topical formulations [7,8,9].

The use of suitable nanocarriers capable of encapsulating the drug in colloidal structures and enhancing its skin permeability could be an effective strategy to improve ibuprofen’s topical bioavailability. Among these, nanogels, commonly defined as aqueous dispersions of nanosized polymeric particles obtained by physical or chemical crosslinking methods, appear particularly attractive in the drug delivery field. In fact, they boast the properties typical of hydrogels, including high water content, which enables rapid diffusion of small molecules, flexibility, biocompatibility and versatility of preparation techniques, and those inherent to their nanoscale size, such as high specific surface area and a large variety of suitably tuneable spatial arrangements [10]. Moreover, compared to other kinds of nanocarriers, such as liposomes or micelles, nanogels are more stable from the physical point of view. However, the main drawback regarding effective use of nanogels is their limited ability to load poorly water-soluble drugs and to adequately regulate and sustain drug release.

An approach frequently used to overcome this disadvantage involves drug incorporation into nanoparticles or vesicles and then loading within the hydrogels [11,12,13]. Another method used to improve the aqueous solubility of hydrophobic drugs is through incorporation into the gels of hydrophilic cyclodextrins (CD) as free molecules, exploiting their complexing/solubilizing ability [14,15,16].

An interesting alternative strategy in this regard is represented by the development of nanogels based on crosslinked CDs, obtained by copolymerization of purposely chemically modified CD monomers with other monomers [17,18,19] or by a CD crosslinking process in an aqueous solution using ethylene glycol diglycidyl ether (EGDE) as the crosslinker [20,21,22,23,24]. In such a way, CD molecules themselves become part of the nanogel’s three-dimensional network, and then they can more effectively improve drug solubility and loading and, additionally, allow a better control of its release. However, chemical modification of CDs can have important drawbacks, including low yield and/or reproducibility of synthesis or presence of residual toxic monomers. On the other hand, the use of chemical crosslinkers, including EGDE, can lead to problems of cytotoxicity [25,26], making long procedures of gel purification necessary to eliminate non-crosslinked residues. Use of a polymeric CD, such as the soluble β-cyclodextrin polymer (EPIβCD), could make it possible to obtain CD-based nanogels without the use of EGDE or other potentially toxic crosslinkers, thus avoiding the time-consuming purification step for their elimination and enabling direct drug loading during nanogel preparation.

Taking into consideration all these premises, the aim of the present study was the development of EPIβCD-based nanogels to improve topical ibuprofen administration by combining the proved CD’s solubilizing effect towards the drug [27] with those of nanogels, including their ability to regulate and prolong drug release and facilitate crossing of tissue barriers [28,29,30].

In order to evaluate the roles of both EPIβCD and the gel preparation method in nanostructures formation and in the drug release behaviour, gel formulations were prepared by two different methods using, alternatively to EPIβCD, a monomeric CD, i.e., hydroxypropyl-β-cyclodextrin (HPβCD), selected as the most effective one among various β-CD derivatives in improving ibuprofen solubility [27].

## 2. Materials and Methods

### 2.1. Materials

Ibuprofen (IBU) was kindly provided by Menarini (L’Aquila, Italy). Hydroxypropyl-β-cyclodextrin (HPβCD) was a gift from Roquette (Lestrem, France); soluble β-cyclodextrin polymer crosslinked with epichlorohydrin (EPIβCD, average MW, 196 kDa) was kindly gifted by Cyclolab (Budapest, Hungary). Dichloromethane, hydroxypropyl methylcellulose (HPMC), and Span 80 (HLB 4.3) were purchased from Sigma (St. Louis, MO, USA). Carbopol 981F (CRP) was purchased from Lubrizol (Wickliffe, OH, USA). Purified water was used (Elix 3 Millipore, Rockville, MD, USA). All other chemicals and solvents were of analytical reagent grade.

### 2.2. HPLC Assay of Ibuprofen (IBU)

The IBU assay was performed by HPLC (Merck Hitachi Elite LaChrom apparatus, Darmstadt, Germany) equipped with an L-2400 UV–vis detector (Hitachi-Science & Technology, Berkshire, UK) and an L-2130 isocratic pump (American Laboratory Trading, East Lyme, CT, USA). A pH 7.4 phosphate buffer solution (PBS)/acetonitrile 50:50 *v*/*v* mixture was used as the mobile phase. A Hibar Purospher^®^ (Merck, Darmstadt, Germany) RP-18e column (150 mm × 4.6 mm, 5 µm pore size) was the stationary phase. UV detection was performed at 222 nm. The injection volume was 20 µL, flow rate 0.9 mL/min, column temperature 40 ± 1 °C. Under these conditions, the IBU retention time was 2.17 ± 0.01 min. The method was validated for linearity (r^2^ = 0.9999), limit of quantification (5.40 µg/mL), and limit of detection (1.60 µg/mL).

### 2.3. Solubility Studies

Solubility studies were carried out by adding an excess amount of IBU to 5 mL of PBS pH 7.4 containing increasing amounts of HPβCD or EPIβCD (10; 15; 20% *w*/*v* for both CDs). The sealed vials were kept under magnetic stirring at 25 °C in a thermostatic bath (25.0 ± 0.1 °C) until equilibrium (24 h). Then aliquots of solution were withdrawn with a filter syringe (pore size 0.45 µm) and analysed by HPLC for drug quantification, as previously described. The absence of interferences due to other components was assessed. A sample containing only IBU and PBS pH 7.4 was analysed as reference. Each experiment was performed in triplicate.

### 2.4. Preparation of CD-Based Gels by Conventional Methods

Two different methods were followed, using HPMC or CRP as the gelling agent.

In the first case, CD (20% *w*/*v*) was dissolved in 25 mL PBS (pH 7.4), and then HPMC was slowly poured up to a final concentration of 2.5% *w*/*w* under stirring and heating at 90 °C for 2 min; after homogeneous dispersion, the system, maintained under stirring, was cooled at room temperature to obtain gelation. In the case of CRP, 20% *w*/*v* CD solution (25 mL) was carefully added to CRP under gently shaking with a glass rod up to a concentration of 2% *w*/*w*; then, after homogeneous dispersion, triethanolamine was added to obtain system gelation.

Drug-loaded hydrogels were prepared using the same procedure by dissolving the drug in the CD solution before the addition of the gelling agent. This procedure assured complete loading of the solubilized drug within the hydrogel matrix.

### 2.5. Preparation of CD-Based Nanogels by the Emulsion-Solvent Evaporation Method

CD-based nanogels were prepared according to the method developed by Moya-Ortega et al. [23] but avoiding the use of EGDE as the crosslinker. The aqueous phase was prepared by dissolving a CD (20% *w*/*v*) in 10 mL PBS (pH 7.4) under magnetic stirring at 60 °C. For the organic phase preparation, Span 80 (1% *w*/*v*) was dissolved in 20 mL dichloromethane. The aqueous phase was allowed to reach room temperature and then added to the organic phase. The mixture was homogenized using an Ultra-Turrax T25 (IKA Werke, Staufen, Germany) at 8000 rpm for 30 s. The obtained W/O emulsion was kept under magnetic stirring at 60 °C for 30 min. Right after that, W/O emulsion was poured into 100 mL water previously heated at 60 °C and then stirred for 180 min until complete evaporation of dichloromethane. The 72 h purification step by dialysis required by the method of Moya-Ortega et al. [23] was not necessary due to the absence of possible toxic residues of unreacted EGDE. Gelation of the systems was finally obtained by slow addition of HPMC (2.5% *w*/*w*) under magnetic stirring and heating at 90 °C for 2 min, followed by cooling at room temperature or, otherwise, by slow addition of CRP (2% *w*/*w*), shaking with a glass rod, and finally adding triethanolamine till the system jelled.

Drug-loaded nanogels were prepared using the same procedure by initially dissolving the drug in the aqueous CD solution before emulsification. This procedure ensured complete loading of the solubilized drug within the hydrogel matrix.

### 2.6. Characterization of CD Nanogels

#### 2.6.1. Dynamic Light Scattering (DLS)

Dynamic light scattering measurements were performed using a Zeta-Sizer Nano ZS90 (Malvern Instruments Ltd., Malvern, UK). Dispersions of gels were diluted in deionized water to avoid scattering phenomena and placed in disposable cuvettes. Each analysis was performed in triplicate at 25.0 ± 0.1 °C.

#### 2.6.2. Differential Scanning Calorimetry (DSC)

DSC analyses of single components and of their physical mixtures at the same *w*/*w* ratio used in the formulations were carried out to evaluate their actual compatibility by a Mettler TA4000 Star^e^ system (Mettler Toledo, Greifensee, Switzerland) equipped with a DSC 25 cell. Accurately weighed samples (MX5 Microbalance, Mettler Toledo, Columbus, OH, USA) were scanned in pierced Al pans at 10 °C/min from 30 to 300 °C under static air.

#### 2.6.3. Determination of pH

The pH values were measured using a Crison 20 pH-meter (Crison Instruments, Barcelona, Spain) after dispersion of an aliquot of the gel in deionized water (1% *w*/*v*). Each analysis was performed in triplicate at 25.0 ± 0.1 °C. The results are the means of three separate measurements.

#### 2.6.4. Determination of Gel Spreadability

Spreadability was evaluated using the properly modified method of Shewaiter et al. [31]. A sample of 0.1 g was placed on a 1 cm diameter circle pre-marked on a glass plate, and then another glass plate was put on it. A weight of 500 g was applied for 5 min on the upper glass plate. Experiments were performed in triplicate at 25.0 ± 0.1 °C. Gel spreadability was determined using the following equation:

% spreadabilty = D_2_/D_1_ × 100

where D_1_ = diameter of the initial circle (1 cm) and D_2_ = diameter of the circle after gel spreading.

#### 2.6.5. Rheological Studies

The rheological behaviour of the formulations was determined by a rotational rheometer (Rheomat 108, Contraves Industrial Products Ltd., Ruislip, UK). Shear flow measurements were carried out using an N3 probe and increasing the shear rate in ascending order from 0 to 150 s^−1^ and then decreasing it from 150 to 0 s^−1^. Each analysis was performed in triplicate on samples equilibrated at 25.0 ± 0.1 °C.

### 2.7. In Vitro Drug Permeation Studies Using Artificial Membranes

In vitro permeation studies of IBU from the examined formulations were performed using Franz diffusion cells (Rofarma, Gaggiano, Italy) with skin-simulating artificial membranes. A fixed amount of gel was placed in the donor compartment. The acceptor medium, consisting of 7 mL PBS (pH 7.4), was maintained at 32 °C (considered as the skin surface temperature) under gentle agitation with a magnetic bar at 50 rpm. Cellulose nitrate membranes (pore size, 0.1 µm) (Sartorius, Göttingen, Germany) impregnated with lauryl alcohol as the lipid phase mimicking the skin barrier [32,33] were employed for the study. At predetermined time intervals, 0.5 mL samples were withdrawn from the receiving compartment and the IBU concentration was assayed by HPLC as described above. A correction for the cumulative dilution due to sample replacement with an equal volume of fresh medium was calculated. All the experiments were performed in triplicate.

### 2.8. Stability Studies

The physicochemical stability of the developed nanogels was checked for 3 months. The prepared samples were kept at 4 °C in sealed containers. At given time intervals, they were checked in terms of visual inspection, pH variation, and rheological behaviour.

### 2.9. Statistical Analysis

Data were statistically analysed by one-way analysis of variance (ANOVA), using the Student–Newman–Keuls comparison post-hoc test to assess the significance of the differences between the groups (GraphPad Prism version 6.0, San Diego, CA, USA); *p*-values < 0.05 or <0.01 were considered significant.

## 3. Results

### 3.1. Solubility Studies

**Figure 1:**
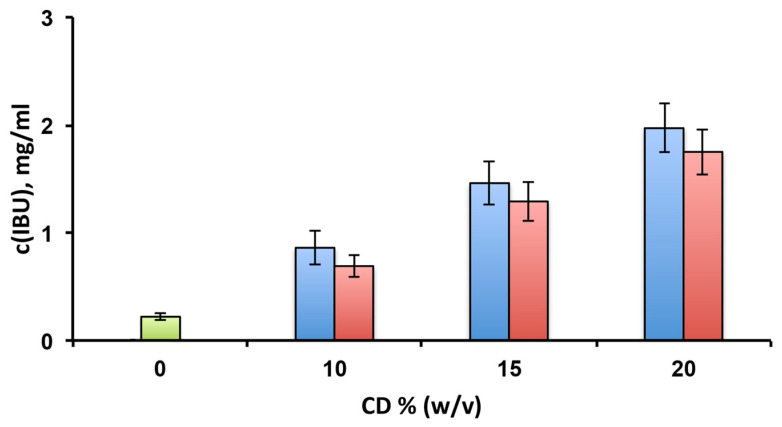
Solubility studies of ibuprofen (IBU) at 25 °C in pH 7.4 phosphate buffer alone (green bar) or in the presence of increasing amounts of HPβCD (blue bars) or EPIβCD (red bars).

Preliminary solubility studies were performed in order to evaluate and compare the solubilising effect towards the drug of the selected CDs (Figure 1). In both cases, the IBU solubility progressively increased with the CD concentration. At the highest concentration of the CDs (20% *w*/*v*), the drug solubility was 1.97 ± 0.23 mg/mL and 1.75 ± 0.21 mg/mL in the presence of HPβCD and EPIβCD, respectively, with the difference between the two tested CDs not statistically significant (*p* > 0.05). These values indicated an about eight times solubility increase compared to free IBU, whose solubility in the same conditions was only 0.22 ± 0.02 mg/mL.

### 3.2. Preparation and Characterization of CD-Based Gels and Nanogels

The main goal of this work was to obtain a nanostructured CD-based gel matrix using a polymeric CD (EPIβCD) and avoiding the use of chemical crosslinking agents, such as EGDE, that could be toxic. The toxicity of EGDE seems to be related to its highly reactive epoxy groups: the presence of unreacted epoxide rings can give rise to toxic effects, both at the systemic level due to their ability to bind with proteins and nucleic acids [34,35] as well as at the dermal level, where they can disrupt cellular membranes of keratinocytes and fibroblast cells [36]. Moreover, two different gelling agents (HPMC or CRP) were investigated in order to evaluate the most effective composition to obtain a nanostructured gel. Furthermore, in order to better point out the double role of EPIβCD as a both drug-solubilizing and nanogel-forming agent, analogous formulations were prepared using a monomeric CD such as HPβCD, which provided a similar solubilizing effect towards IBU. In addition, all these CD-based gel formulations were also prepared by conventional methods so that we can also separately evaluate the influence of the preparation procedure on the formulation properties and performance. All the resulting products, after suitable dilution, were first analysed by DLS.

**Figure 2:**
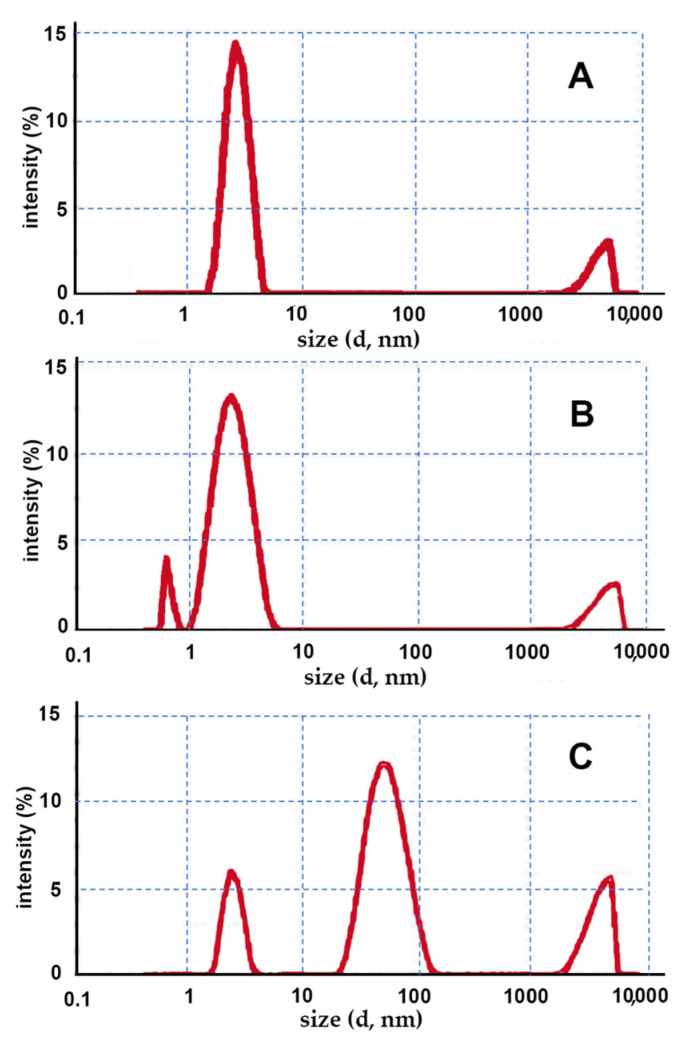
Intensity fraction size distribution graphs of the gels prepared by conventional methods obtained by DLS; the following CD–gelling agent combinations were used: (**A**) HPβCD–HPMC; (**B**) HPβCD–CRP; (**C**) EPIβCD–CRP.

Conventional methods for preparing classic gel formulations are very simple, only requiring addition to the aqueous CD solution of a gelling agent (HPMC or CRP), followed by system gelation, obtained by varying the dispersion temperature or pH, respectively. In Figure 2, the size distribution graphs obtained by DLS for the gels prepared using traditional methods are provided.

In the case of the gels containing HPβCD (Figure 2A,B), regardless of the type of the gelling agent used, an almost unimodal size population was observed, with the mean size around approximately 3 nm, which was reasonably attributable to CD monomers. Instead, the low-intensity band peaked over 5000 nm was reasonably due to the presence of traces of clusters of gel not homogeneously dispersed in the sample. As expected, no formation of nanostructures was observed.

On the contrary, the sample with the EPIβCD–CRP combination (Figure 2C) showed an intense size population peaked at around 60 nm, likely attributable to a nano-matrix structure formed by the polymeric CD. These results seem to indicate that under the used experimental conditions, the polymeric chains of EPIβCD were able to spontaneously assemble in the aqueous medium, giving rise to the formation of nanosized aggregates. On the other hand, the very poorly intense small size population peaked at around 3 nm could be explained by the presence of traces of residual monomeric CDs, while the band peaked around 5000 nm could be due (as in the previous cases) to the presence of traces of clusters of undispersed gel in the sample.

Unexpectedly, in the case of the EPIβCD–HPMC system, some kind of incompatibility occurred during sample preparation. In fact, after the addition of the gelling agent to the CD solution and 2 min heating at 90 °C, a nonuniform gelation of the system occurred during the subsequent cooling under stirring at room temperature, resulting in an upper part rich in HPMC, which showed a gelled texture, and a lower part remaining in the liquid state.

DCS analyses were performed to assess the compatibility between the formulation components. With this aim, thermal curves of the single pure components were compared with those of their corresponding physical mixtures prepared at the same *w*/*w* ratios used in the formulations. The DSC curves of the various physical mixtures resulted in all cases in the simple superimposition of those of the corresponding single components, thus indicating the absence of solid-state interactions and then their compatibility (data not shown).

This result proved that the observed phenomenon of uncompleted irregular gelation of the sample containing the EPIβCD–HPMC combination has to be attributed to the problem of thermodynamic incompatibility occurring during gel preparation, giving rise to the observed segregating phase separation; this was probably related to the absence of attractive forces between HPMC and EPIβCD, joined to the low energy provided to the system by magnetic or manual stirring during conventional gel preparation methods [37].

The same formulations were also used to prepare nanogels by the emulsion/solvent evaporation technique using the suitably modified method proposed by Moya-Ortega et al. [23]. In particular, it was possible to avoid the long purification step that required three days of dialysis necessary to eliminate impurities due to unreacted residues of the chemical crosslinker (EGDE).

**Figure 3:**
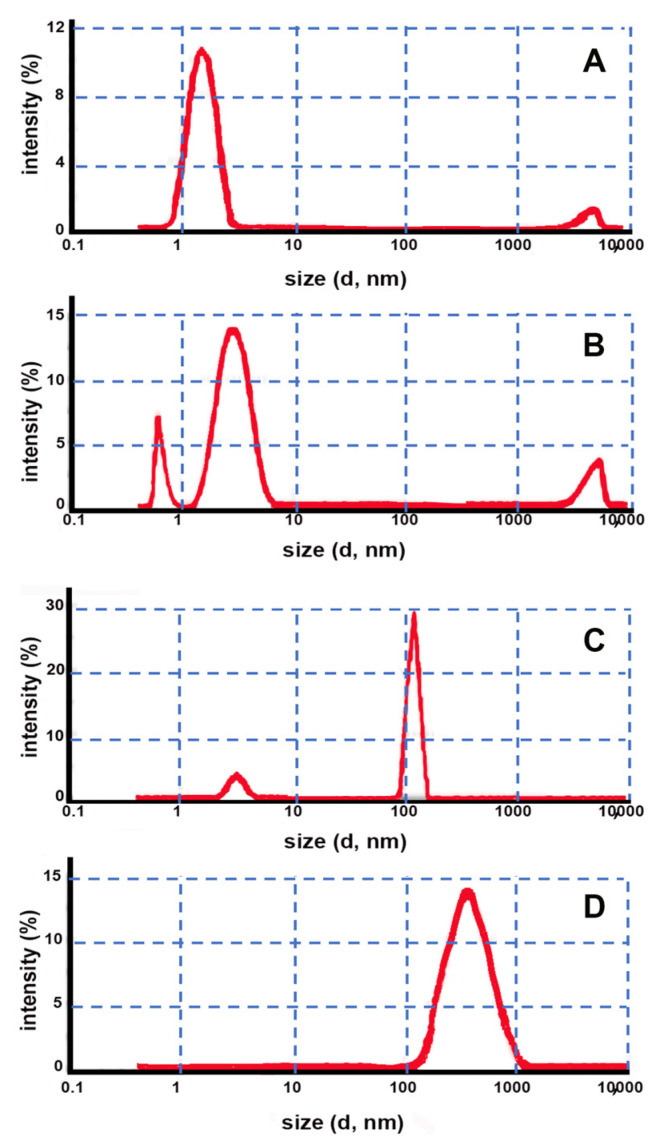
Intensity fraction size distribution graphs obtained by DLS of gels prepared by the emulsion method using the following CD–gelling agent combinations: (**A**) HPβCD–HPMC; (**B**) HPβCD–CRP; (**C**) EPIβCD–HPMC; (**D**) EPIβCD–CRP.

As can be seen in Figure 3, in this case, the DLS analysis of both samples containing HPβCD in combination with both HPMC and CRP (Figure 3A,B) also did not show the presence of a nanostructured gel matrix similarly to what was previously observed for the corresponding gel prepared by the conventional method. In fact, only an intense small size population with a peak at around 3 nm attributable to CD monomers and a low intensity band around 5000 nm attributable to traces of small agglomerates of not well-dispersed gel in the sample were present.

On the contrary, both formulations containing EPIβCD behaved like a nanostructured gel, exhibiting practically only a very sharp peak at around 122 nm when using HPMC as the gelling agent and a single size population peaked around 400 nm when using CRP (Figure 3C,D).

Considering these data, it can be concluded that the use of a monomeric CD, such as HPβCD, without any crosslinker (such as EGDE) made it not possible to obtain a nanogel structure regardless of both the type of the preparation method and the gelling agent used, thus confirming the results of Moira-Ortega et al. [23]. On the contrary, the use of a polymeric CD, such as EPIβCD, allowed the formation of a nanostructured gel matrix without the need for adding any chemical crosslinker. In particular, using CRP as the gelling agent, the formation of nanosized aggregates was obtained using both of the considered preparation methods; instead, in the presence of HPMC, nanogel formation took place only when using the emulsion-solvent evaporation method, while phase separation problems were encountered under the experimental conditions of the conventional method, probably due to the lower energy provided to the system, as discussed above.

Interestingly, the size distribution of the nanogels was strongly affected by both the kind of the gelling agent used and the gel preparation method, ranging from 60 nm for the nanogel obtained with the conventional method and CRP as the gelling agent to 120 nm for that obtained by the emulsion-solvent evaporation method and HPMC as the gelling agent and up to 400 nm for that obtained by the emulsion-solvent evaporation method but with CRP as the gelling agent.

All the three kinds of nanogel formulations obtained using EPIβCD were selected for a further characterization and compared with three commercial gels, all containing IBU as lysine salt: Vegetallumina Antidolore^®^, Dolofast^®^, and Lasonil Antidolore^®^.

**Figure 4:**
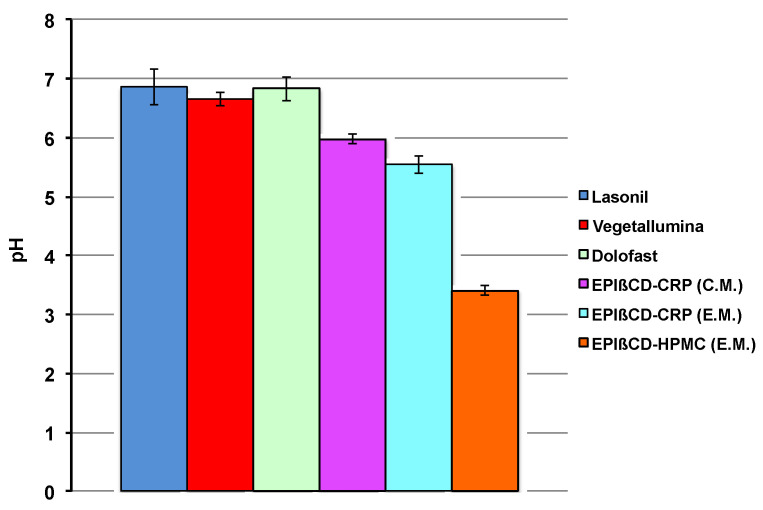
Values of pH of the examined commercial formulations and of the developed nanogel formulations based on EPIβCD in combination with CRP or HPMC obtained by the conventional method (C.M.) or the emulsion method (E.M.).

It is well-known that the skin has several properties and functionalities, including thermoregulation, a highly selective barrier effect, protection against bacteria proliferation, etc. Among these, the acidic pH of the skin surface has been indicated as an important factor in maintaining the stratum corneum homeostasis and the skin barrier function. It has been found that it varies from 4 to 6 depending on the body zone [38], and then there is a broad general agreement that the optimal pH of topical formulations should fall within such a range [39]. The pH values of all the investigated formulations are shown in Figure 4.

All the commercial products showed similar pH values, near to the neutrality, which can be attributed to the presence of IBU as a salt of the basic aminoacid lysine. Both EPIβCD-based nanogels prepared with CRP as the gelling agent satisfied the required optimal pH values, which ranged from 5.55 to 5.98, owing to the presence of triethanolamine added for obtaining system gelation; on the contrary, the sample with HPMC showed a pH around 3.5, attributable to the acidic character of IBU.

**Figure 5:**
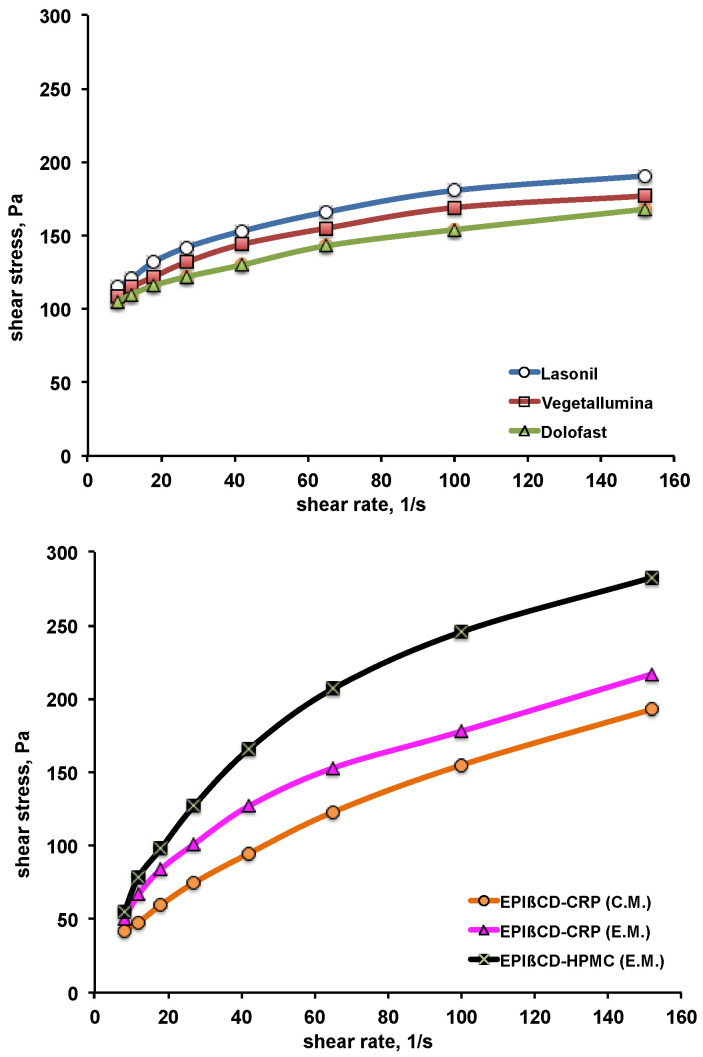
Rheological behaviour of the examined commercial formulations (**top**) and of the developed nanogel formulations based on EPIβCD in combination with CRP or HPMC obtained by the conventional method (C.M.) or the emulsion method (E.M.) (**bottom**).

The rheological behaviour of the formulations was evaluated, as it is correlated to their consistency and spreadability on skin surface. The obtained plots of the shear stress as a function of increasing shear rate (up-curves) are shown in Figure 5. All the commercial products exhibited a non-Newtonian plastic behaviour, typical of the large porous structure of conventional gels, exhibiting yield point values ranging between 105 and 115 Pa. The developed nanogels showed instead a pseudoplastic behaviour, without an appreciable yield point. The viscosity values at the shear rate of 150 s^−1^ were around 1.2–1.4 Pa·s for all the formulations, with the exception of the EPIβCD-based nanogels containing HPMC, which showed the highest viscosity value, around 1.8 Pa·s. All the formulations exhibited a shear rate dependence anyway, with shear tinning behaviour, i.e., a rapid decrease in viscosity as the shear increased and consequent favourable fluidity. The absence of thixotropy was assessed since all the down-curves (obtained by decreasing the shear rate from 150 to 0 s^−1^) were perfectly superimposed to the corresponding up-curves (data not shown), thus indicating that structure regeneration was not time-dependent. Based on rheological measurements, all the formulations were suitable for dermal use.

The possible influence of drug presence on the rheological behaviour of the different formulations was evaluated. Unloaded gels analysed in the same conditions showed superimposable profiles to those of the corresponding loaded ones, thus excluding any effect of drug presence.

**Figure 6:**
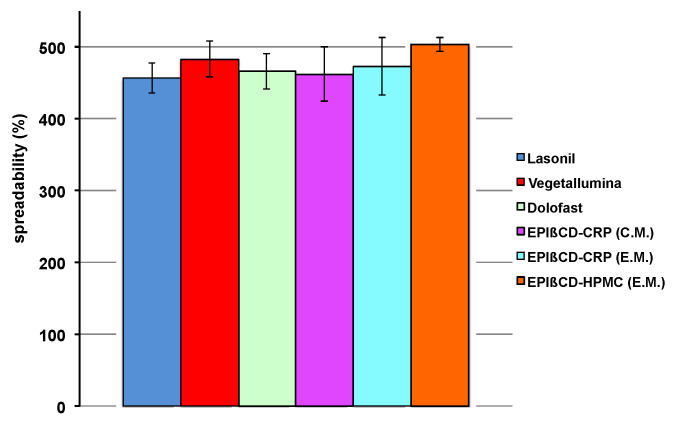
Spreadability values of the examined commercial formulations and of the developed nanogel formulations based on EPIβCD in combination with CRP or HPMC obtained by the conventional method (C.M.) or the emulsion method (E.M.).

Another essential feature of a topical formulation is its spreadability. In fact, it should be easily spreadable, without having to apply too much drag, but at the same time it should not be too liquid, leading to product leakage. The values of spreadability shown in Figure 6 indicate that all the developed nanogels exhibited suitable spreadability comparable to that of the commercial products.

### 3.3. In Vitro Permeation Studies

Skin permeation studies are of basic importance in the development of dermal and transdermal formulations. However, due to a series of economic, practical, and ethical reasons, in vivo experiments on humans are not feasible during the initial screening at the level of pre-formulation studies. Therefore, alternative faster and cheaper assays based on easily accessible and well-reproducible in vitro models simulating skin behaviour are essential to test the performance of dermal products in pre-formulation stages [40]. Among these, Franz diffusion cells are a method largely used for assessing in vitro permeation of drugs from excised animal skin or synthetic membranes [41,42]. In particular, the use of artificial membranes treated with lipids seems to be a valid and reproducible method to mimic the skin barrier properties of the stratum corneum, avoiding the problems of high variability and ethical issues related to biological skin samples and allowing an effective preliminary screening and comparison of drug permeability from different formulations [32,33,43,44]. On the other hand, simple diffusion studies using an untreated non-barrier cellulose membrane may only reflect drug release and not its permeation across the skin, thus providing limited insight and resulting in a worse prediction of the actual performance of topical or transdermal formulations [41,42,45]. In this work, artificial cellulose nitrate membranes soaked with lauryl alcohol previously developed as simulating the epidermal barrier [32,33] were employed.

**Figure 7:**
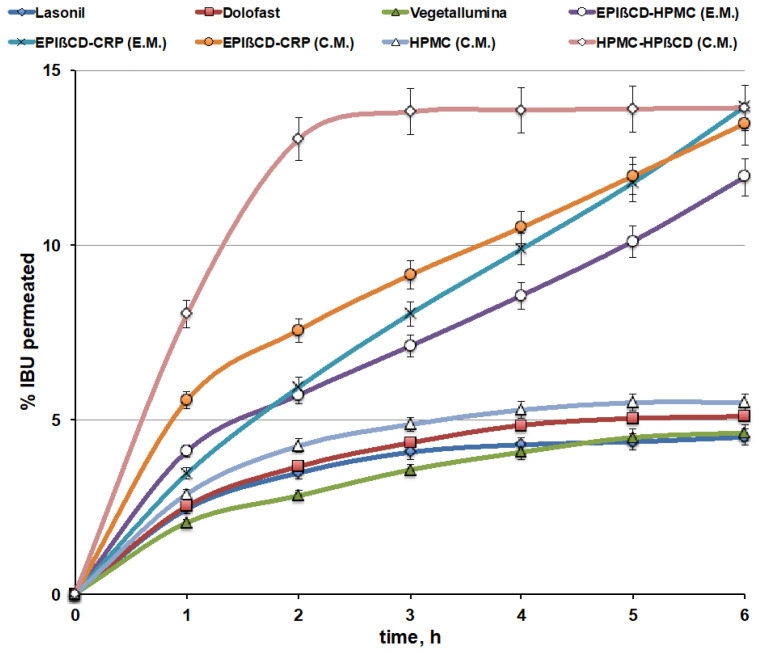
Drug permeation curves of the nanogel formulations based on EPIβCD in combination with CRP or HPMC obtained by the conventional method (C.M.) or the emulsion method (E.M.) compared with those from commercial gel formulations and a reference HPMC gel obtained by the conventional method (C.M.), containing HPβCD or not.

The permeation profiles of IBU from the EPIβCD-based nanogels (through artificial lipophilic membranes) were compared to those of the commercial products (Figure 7). Moreover, in order to better evaluate the double role of the polymeric EPIβCD in the formulation, i.e., its ability to host the drug within its cavities, thus allowing to improve the loading of a hydrophobic drug into a hydrophilic gel and better control its release, combined with its proved capacity to form a nanostructured gel network, which should further concur to modulate and sustain drug release, the drug permeation behaviour of conventional HPMC gels, containing HPβCD or not, was also evaluated.

The drug permeation profiles from the commercial gel products were very similar to each other, showing a very low percentage of the permeated drug, with an almost “plateau phase” after about 3 h and in no case exceeding 5% after 6 h. On the contrary, the developed EPIβCD-based nanogel formulations showed a clearly better behaviour, with a progressively increasing percentage of the permeated drug over time, reaching almost 15% after 6 h. The slopes of the drug permeation curves from nanogels, index of the drug permeation rate, were from 2.4 up to 3.5 times higher than those from commercial gels, and the progressive increase in the cumulative amount permeated lasted longer than the considered 6 h. For this reason, permeation experiments on such formulations were extended up to 24 h. It was found that the uptrend behaviour of the permeation curves was maintained for over 12 h, followed by a gradual decrease in the drug permeation rate, reaching about 38, 35, and 28% of the drug-released amount after 24 h for the EPIβCD–CRP (E.M.), EPIβ–CRP (C.M.), and EPIβCD–HPMC (E.M.) nanogel formulations, respectively.

Examination of the overall results of the permeation studies indicated that the better performance, with the lowest initial burst effect and the highest slope of the permeation curve, was provided by the nanogel based on the EPIβCD–CRP combination, prepared by the emulsion method (EPIβCD–CRP (E.M.)), which presented the greatest size distribution of the nanogels (400 nm). On the contrary, the highest initial burst effect was observed for the nanogel with the same composition but obtained by the conventional method (EPIβCD–CRP (C.M.)), which showed the smallest size distribution of the nanoaggregates (60 nm). Finally, the nanogel based on the EPIβCD–HPMC combination showed the lowest drug permeation rate, probably due to its highest viscosity, as observed in rheological studies.

On the other hand, the simple HPMC-based gel prepared by the conventional method showed a permeation profile similar to those of the commercial gel products, rapidly reaching a plateau after about 2 h and achieving only slightly more than 5% of the drug permeated after 6 h. Interestingly, the corresponding gel containing 20% of HPβCD showed a marked increase in the percentage of the permeated drug, reaching about 15% after only about 2.5 h, but this fast initial phase was immediately followed by a plateau. These results point out the favourable effect of CD presence in increasing the drug’s aqueous solubility and then drug release and permeation, but at the same time show that simple addition of a nonpolymeric CD to a conventional gel was not able to provide a controlled and sustained drug release since it did not modify the gel structure, as shown by DLS analysis. On the contrary, both these goals, i.e., improved drug solubility and sustained release, were achieved in the case of the EPIβCD-based nanogels, where the encapsulation of the drug within the cavities of the polymeric βCD combined with the colloidal structure of the nanogel provided by the polymeric CD allowed not only to increase drug solubility and release, but also to better control and prolong its release over time. On the other hand, the initially slower release observed in comparison with the gel containing the monomeric HPβCD confirmed the actual sustained-release ability of the nanogel, in agreement with the results of Moya-Ortega et al. [23].

In order to obtain insight about the kinetics of the drug permeation from the different formulations, according to analogous studies [41,43,44], permeation data were fitted to the main mathematical kinetic models, namely zero-order, first-order, Higuchi, and Korsmeyer–Peppas [46]. The best-fitting model was chosen on the basis of the highest correlation coefficient value (R^2^), as determined by linear regression. The obtained results are summarised in Table 1.

Regarding the commercial gel formulations, the model that better fitted the permeation data was in all cases the Korsmeyer–Peppas equation, with values of the release exponent n below 0.5, suggesting that the drug release was governed by the combination of two diffusion processes through both the gel matrix and the water-filled pores [43,47].

On the contrary, in the case of nanogel formulations, release data fitted equally well with zero-order, first-order, and Korsmeyer–Peppas models, emphasizing that, in this case, more mechanisms simultaneously concur in determining the drug permeation behaviour due to the presence of the polymeric EPIβCD and its ability to interact with the drug, increasing its solubility and affecting its release, and to generate a nanostructured gel capable of providing sustained release. The values of the release exponent n ranged between 0.6 and 0.7, indicative of an anomalous (non-Fickian) transport mechanism [48]. On the other hand, both the type of the gelling agent (CRP or HPMC) and the nanogel preparation method did not substantially affect the kinetic behaviour.

### 3.4. Stability Studies

The stability of the developed nanogel formulations was monitored by visual inspection, rheological behaviour, and pH control during the 3 months of storage at 4 °C. The visual inspection evidenced the absence of the formation of mould or of sedimentation, creaming, or phase separation phenomena. The rheograms of all the stored nanogels showed exactly the same profiles as those of the corresponding fresh samples. Moreover, no significant variations of pH were recorded during the whole storage period. These results were considered indicative of a good physicochemical stability of the developed formulations.

## 4. Conclusions

In this work, a CD-based nanogel aimed to improve cutaneous IBU delivery was successfully developed without the addition of any potentially toxic crosslinking agent, such as EGDE, thanks to the use of the soluble βCD/epichlorohydrin polymer EPIβCD.

The proposed strategy provided three main benefits, namely (1) avoidance of the long dialysis purification procedures needed to eliminate impurities due to unreacted reagent molecules; (2) direct loading of the drug during the nanogel preparation, unlike in the other methods requiring its successive incorporation into the preformed nanogels [22,23,24,49], thus enabling the entrapment within the nanogel structure of the whole drug amount dissolved in the CD solution before the gelling agent addition; (3) faster and easier nanogel preparation procedure, which facilitates its future industrial scaleup.

It has been proven that the role of EPIβCD in the formulation was crucial, since it not only acted as a complexing/solubilizing agent towards the hydrophobic poorly soluble drug, thus enabling an increase in both its loading and release, but it was also essential to allow the formation of a nano-matrix structure capable of suitably sustaining drug release over time.

DLS analysis showed that the presence of EPIβCD enabled the formation of nanostructured gels regardless of the preparation method and without the need in a crosslinking agent. The size distribution of the formed nanoaggregates ranged from 60 up to 400 nm depending on the type of the gelling agent used and the gel preparation method. On the other hand, the replacement of EPIβCD with a monomeric βCD, such as HPβCD, did not allow in any case the nanogel formation regardless of the preparation method and the gelling agent used.

The developed EPIβCD-based nanogels showed better drug release and permeation profiles through artificial skin-simulating lipophilic membranes compared to both the reference gel formulation prepared without a CD and to the commercial IBU gel formulations currently on the market. In fact, EPIβCD not only increased drug solubility and enhanced the drug permeation rate, but also acted as nano-matrix forming, thus providing sustained drug release over time. The degree of increase in the drug permeation rate (from 2.4 up to 3.5 times) seemed to be related to the size of nano-aggregates. On the other hand, simple addition of HPβCD to the reference gel formulation gave rise to a strong increase in drug solubility and initial release from the gel, proving to be useful for obtaining an intense and fast but less lasting effect; in fact, it was not able to provide sustained drug release due to its inability to form a nano-matrix structure controlling and modulating drug diffusion.

## Figures and Tables

**Table 1:** Kinetic study of IBU permeation through artificial membranes from the examined commercial formulations and from the developed nanogels based on EPIβCD in combination with CRP or HPMC prepared by emulsion-solvent evaporation (E.M.) or by conventional (C.M.) methods.

Formulation	Zero-Order	First-Order	Higuchi	Korsmeyer–Peppas	
	R^2^	R^2^	R^2^	R^2^	n
Lasonil	0.8138	0.8157	0.8988	0.9343	0.4
Vegetallumina	0.8517	0.8530	0.9869	0.9929	0.3
Dolofast	0.8708	0.8728	0.9413	0.9638	0.4
EPIβCD–CRP (E.M.)	0.9982	0.9985	0.9890	0.9985	0.6
EPIβCD–CRP (C.M.)	0.9936	0.9985	0.9890	0.9985	0.7
EPIβCD–HPMC (E.M.)	0.9933	0.9924	0.9771	0.9902	0.6
